# Contraception and abortion in times of crisis: results from an online survey of Venezuelan women

**DOI:** 10.3389/fgwh.2023.1189706

**Published:** 2023-09-18

**Authors:** Bianca M. Stifani, Genesis Luigi, Genevieve Tam, Nina Zamberlin, Giselle Carino, Susana Medina-Salas, Tamara Fetters, Roopan Gill

**Affiliations:** ^1^ Department of Obstetrics & Gynecology, New York Medical College/Westchester Medical Center, Valhalla, NY, United States; ^2^ Vitala Global, Vancouver, BC, Canada; ^3^ Fòs Feminista, New York, NY, United States; ^4^ Ipas, Chapel Hill, NC, United States; ^5^ Department of Obstetrics & Gynecology, University of Toronto, Toronto, ON, Canada

**Keywords:** abortion, contraception, Venezuela, survey, social media, humanitarian crisis, sexual and reproductive health, digital health post-abortion care

## Abstract

**Introduction:**

In the last decade, Venezuela has experienced a complex humanitarian crisis that has limited access to healthcare. We set out to describe Venezuelan women's experiences accessing sexual and reproductive health services, including abortion, which is heavily restricted by law.

**Methods:**

We fielded an online survey in July of 2020 among Venezuelan women recruited through social media advertisements. We conducted descriptive statistical analyses using Excel and STATA SE Version 16.0.

**Results:**

We received 851 completed survey responses. Almost all respondents experienced significant hardship in the last year, including inflation (99%), worries about personal safety (86%), power outages (76%), and lack of access to clean water (74%) and medications (74%). Two thirds of respondents used contraception in the last two years, and almost half (44%) of respondents had difficulty accessing contraception during that same time period. About one fifth of respondents reported having had an abortion; of these, 63% used abortion pills, and 72% reported difficulties in the process. Half of those who had an abortion did it on their own, while the other half sought help – either from family members or friends (34%), from providers in the private health sector (14%), or from the Internet (12%).

**Conclusions:**

Venezuelan women who responded to our survey describe a harsh context with limited access to sexual and reproductive health services. However, they report relatively high rates of contraceptive use, and abortion seems to be common despite the restrictive legal setting.

## 1. Introduction

For the last decade, Venezuelans have lived in crisis. In the early years of President Hugo Chavez' rule, the country experienced an oil-fueled economic boom, with promising results at the public health level, such as rising life expectancy and decreased infant mortality rates (1). When oil prices began to fall in 2008, the country's economy crashed, leading to hyper-inflation and a fall in the gross domestic product. Lack of foreign currency needed to import necessary goods and supplies led to a shortage of basic necessities – including fuel, medications and healthcare supplies (1). Many Venezuelan healthcare workers emigrated, including about half of the country's physicians, further contributing to a complete collapse of the country's healthcare system (2).

Human rights organizations have described the Venezuelan context as a “complex humanitarian emergency,” a situation in which a considerable breakdown of authority leads to human suffering on a major scale (3). According to the fragile states index, Venezuela was among the top five countries that worsened in the last decade (4). A report published in 2019 based on data collected by non-governmental organizations showed that almost all households experienced food insecurity, and rates of hospital admissions for childhood malnutrition were rising rapidly (5).

Accessing sexual and reproductive health (SRH) services in this context is challenging. For many, purchasing contraceptives can be cost-prohibitive (6). These are often unavailable due to chronic supply shortages, and are considered to be luxury items purchased on clandestine markets (6). Access to abortion is restricted by Venezuelan law, which only allows it when a woman's life is at risk (7). Although recent official government data are lacking, news organizations and civil society groups in Venezuela have described a crisis in maternal health (8). From 2015 to 2016, the last years for which official data are published, maternal deaths increased by 65% (from 456 to 756) (9). Maternal complications from unsafe abortion such as hemorrhage and infection accounted for 7% of maternal deaths reported in Venezuelan health facilities in a six-month period in 2018 (10).

News and humanitarian reports have described the challenges of accessing SRH services in Venezuela, but few if any research studies have broached this topic. We aimed to describe Venezuelan women's successes and challenges in accessing abortion and contraception in this complex humanitarian context.

## 2. Methods

This is a cross-sectional survey study. We conducted this survey as part of a three-phase user-centered design project aimed at developing a digital tool for self-managed abortion in Venezuela. For Phase 1 of this project, we collected quantitative and qualitative data to understand how women access sexual and reproductive health services in Venezuela, as well as their preferences for receiving information about these topics. Here, we report on the quantitative results of this phase of the project.

The research team consisted of international obstetrics & gynecology clinicians and researchers, women's rights activists, and local sexual and reproductive healthcare providers. We collaborated to design a survey, which we pilot-tested with Venezuelan women and modified accordingly.

We fielded the online survey using social media platforms. In partnership with a specialized media firm, we disseminated social media advertisements through the Facebook and Instagram accounts of PLAFAM (Asociación Civil de Planificación Familiar), a sexual and reproductive healthcare organization in Venezuela that is a partner of Fòs Feminista.

We only included completed responses in the analysis. We used Microsoft Excel and Stata SE Version 16.0 to conduct descriptive statistical analyses of survey responses.

The Allendale Institutional Review Board approved this study.

## 3. Results

We advertised the survey during six days in July of 2020, and received a total of 851 completed survey responses. Table 1 shows the demographic characteristics of survey respondents. Most were young women (27% were 19–25, and 36% were 26–35). Almost half (360, 42%) of respondents had completed university and a third (279, 32.8%) had some university education. Most respondents had encountered social and economic challenges in the past year, with the most common being inflation (99%), worrying about personal safety (86%), and power outages (76%).

**Table 1:** Characteristics of individuals who responded to an online survey conducted in 2020 about sexual and reproductive health in Venezuela.

Gender Identity	*n*	% (*n* = 851)
Female	847	99.5
Other	4	0.5
Age		
14–18	44	5.2
19–25	229	26.9
26–35	304	35.7
36–45	211	24.8
>46	63	7.4
Country of birth		
Venezuela	842	98.9
Other	9	1.1
Education		
Completed primary school or less	3	0.4
Some secondary school	60	7.1
Completed secondary school	149	17.5
Some university	279	32.8
Completed university	360	42.3
Occupation		
Employed full time or part time	373	43.8
Self-employed	173	20.3
Home maker	71	8.3
Student	98	11.5
Unemployed/looking for work	117	13.7
Other	19	2.2
Receiving financial assistance from government		
Yes	132	15.5
No	719	84.5
Monthly income		
One minimum wage or less	203	23.9
Around two minimum wages	153	18.0
More than two minimum wages	204	24.0
No income	154	18.1
Prefer not to answer	137	16.1
Challenges encountered in the past year*		
Inflation	844	99.2
Worried about personal safety	732	86.0
Power outages	645	75.8
Lack of access to clean water	630	74.0
Lack of access to medications	630	74.0
Lack of access to the Internet	568	66.7
Lack of access to basic medical care	568	66.7
Lack of gasoline	522	61.3
Lack of food	481	56.5
Lack of cooking gas	421	49.5
Difficulty accessing housing	359	42.2
Migration	136	16.0

* Many respondents selected more than one challenge.

### 3.1. Contraception

When asked about contraceptive use, approximately two thirds of respondents (565, 66.4%) reported having used a contraceptive method in the last two years. The most popular methods were the male condom (240, 43.9%) and the implant (212, 37.5%), followed by contraceptive pills (151, 26.7%) and the IUD (141, 25.0%). Most respondents obtained contraceptives from PLAFAM (56.3%) or from a pharmacy (40.7%). Almost half of respondents who used contraception (247, 44.0%) had difficulties accessing contraceptive methods in the last two years. The most common difficulties experienced were high cost (239, 96.8%), lack of availability of their preferred method (193, 78.1%), and transportation issues (77, 31.2%). Table 2 shows contraceptive use and difficulty accessing contraception.

**Table 2:** Contraceptive access, use and challenges among individuals who responded to an online survey conducted in 2020 in Venezuela.

Have used contraception in the last two years?	*n*	% (*n* = 851)
Yes	565	66.4
No	286	33.6
If yes, which methods did you use? *	*n*	% (*n* = 565)
Male condom	240	42.5
Implant	212	37.5
Contraceptive pill	151	26.7
IUD	141	25.0
Pull-out method	132	23.4
Emergency contraception	76	13.5
Natural methods	24	4.25
Tubal ligation	18	3.2
Other	6	1.1
Where do you usually obtain contraceptive methods? *	*n*	% (*n* = 565)
At PLAFAM	318	56.3
At the pharmacy	230	40.7
From friends, family, or a partner	63	11.2
At a public hospital or clinic	59	10.4
From a private doctor	54	9.6
Online	37	6.5
From street vendors	21	3.7
Other	16	2.8
Have you had difficulty accessing contraception in the last two years?	*n*	% (*n* = 565)
Yes	247	44.0
No	318	56.0
Which difficulties did you have accessing contraception? *	*n*	% (*n* = 247)
High cost	239	96.8
Method not available	193	78.1
Transportation issue	77	31.2
No staff at the health post	23	9.3
Needed parental authorization	12	4.9
Needed partner permission	7	2.8
Was denied the method	9	3.6
I got a fake method	4	1.6
Other	18	7.3

PLAFAM, asociación civil de planificación familiar.

* Some respondents selected more than one method, location, or difficulty.

### 3.2. Abortion

Almost half of survey respondents (364, 42.8%) experienced an unplanned pregnancy in their lifetime, and one fifth had an abortion at some point in their life (169, 19.9%). Two thirds of respondents (563, 66.2%) reported they knew a friend or family member who has had an abortion. Of the 169 respondents who had an abortion, roughly two thirds did so using abortion pills (106, 62.7%), while about one third had a procedure performed by a physician (61, 36.1%). Other abortion methods such as herbal remedies and procedures performed by non-physicians were less frequent. Some respondents selected more than one abortion method. Approximately half of respondents who had an abortion experienced difficulties accessing one, with the most common difficulty being fear that something bad would happen (82, 48.5%). Half those who had an abortion (via any method) sought help from other people, while the other half did not. Table 3 shows access to abortion and difficulties experienced in this process.

**Table 3:** Access to abortion among individuals who responded to an online survey conducted in 2020 in Venezuela.

Have you ever had an unplanned pregnancy?	*n*	% (*n* = 851)
Yes	364	42.8
No	487	57.2
Do you know a friend or family member who has had an abortion?	*n*	% (*n* = 851)
Yes	563	66.2
No	288	33.8
Have you ever had an abortion?	*n*	% (*n* = 851)
Yes	169	19.9
No	682	80.1
What did you use to have an abortion? *	*n*	% (*n* = 169)
Took abortion pills	106	62.7
A physician did a surgical procedure on me	61	36.1
Took herbal remedies	21	12.4
Nothing (spontaneous abortion)	8	4.7
A person who was not a physician did a surgical procedure	2	1.2
Did you have any difficulty in getting an abortion?	*n*	% (*n* = 169)
Yes	121	71.6
No	43	25.4
If yes, which difficulties? *	*n*	% (*n* = 169)
No difficulties	83	49.1
I was afraid something bad would happen	82	48.5
I didn't know who to ask for help	33	19.5
I didn't know what to do	32	18.9
It was very expensive	31	18.3
I was scared of getting arrested	25	14.8
Couldn't find the pills	17	10.1
Lack of privacy	8	4.7
I was far along in the pregnancy	7	4.1
Transportation problems	4	2.4
Did anyone help you get an abortion?	*n*	% (*n* = 169)
Yes	82	48.5
No	82	48.5
Who or what helped you get an abortion? *	*n*	% (*n* = 169)
Friend or family member helped	58	34.3
I looked online	21	12.4
Provider in the private sector helped	23	13.6
Other	15	8.9
Feminist group helped via phone	11	6.5
Provider in the public health system helped	8	4.7
International NGO helped	5	3.0
Feminist group helped in-person	4	2.4
Community organization helped	1	0.6

* Some participants selected more than one answer.

## 4. Discussion

Results from this online survey of Venezuelan women show that although almost two thirds of respondents used some form of contraception in the two years prior to the survey, almost half of them had problems accessing contraceptive methods in the context of the country's ongoing humanitarian crisis. This finding aligns with existing reports from newspapers and nonprofit organizations (2, 6). A 2019 report published by a coalition of civil society groups reported on scarcity of contraceptives in pharmacies in five cities in Venezuela. They reported that in December of 2018, for example, 75% of the pharmacies queried did not have any contraceptive pills available. The scarcity index was even higher for other methods, including injectables and long-acting reversible methods (11).

We found that the male condom was the most common method used but are unable to tease out whether this was used alone or with other methods. Condoms are easy to access at PLAFAM (for free or subsidized) or from pharmacies. This may make them more easily accessible than other methods. It is interesting to note that the contraceptive implant was one of the most common methods used by our survey respondents, despite it being very difficult to find in Venezuelan pharmacies (11). This is likely because our recruitment strategy targeted social media users who had visited the PLAFAM page. PLAFAM is one of the few organizations that provides free or low cost contraceptive implants in Venezuela (2), and half of our respondents obtained their contraceptive method there. The contraceptive implant is the method most provided at PLAFAM, followed by intrauterine devices.

Another important finding of this study is that despite the near total ban on abortion in Venezuela, a fifth of respondents reported they had an abortion sometime in their life. This is in line with Latin American and the Caribbean regional estimates from the Guttmacher Institute regarding the share of unintended pregnancies that end in abortion, which is approximately 46%. In 2015–2019, there were almost 8 million unintended pregnancies and almost 4 million abortions (12). Banning abortion does not prevent it, but rather increases the proportion of abortions that are unsafe (13). We note that only 14.8% of our respondents who had an abortion reported fear of being arrested as a difficulty in accessing abortion. Thus, there seemed to be relatively little fear of legal prosecution reported by our respondents despite the restrictive legal landscape.

In our study, abortion pills were the most common method used for abortion (106, 62.7%). We did not ask about specific pill regimens used, but we assume that most individuals used misoprostol alone. Mifepristone is not registered in Venezuela, though it can sometimes be obtained through international organizations. For the third of respondents who had abortions performed by a physician, we are unable to delineate whether these were legal abortions under the exception to save the life of the woman or clandestine induced abortion procedures. Some may have been provision of post-abortion care after incomplete abortions induced with medications. In any case, it is interesting to note that physicians are involved in a large proportion of the abortions reported by our respondents.

Our findings add to the current state of knowledge about abortion in Venezuela, which comes primarily from reports of abortion hotlines run by feminist accompaniment groups. A 2019 report published by Faldas-R, a group that runs an abortion support hotline, called abortion a “daily occurrence” in Venezuela, supporting our finding of abortion being relatively common (14). This group reported a 40% increase in calls to their hotline from 2018 to 2019, suggesting that the incidence of abortion may be increasing with the worsening economic crisis. The authors of a qualitative study that examined abortion hotlines in five countries describe one hotline in Venezuela, which received approximately 450 calls per month (15). While these collectives serve an important role in helping women access safe abortion, less than ten percent of our respondents who had an abortion sought the help of a women's group, either in-person or via phone. Most went to friends, family members, and private physicians – thus suggesting that abortion is even more common than the estimates based on hotline calls.

Our study has several limitations. First, it is a survey study and as such it is subject to response bias. Because the survey was hosted on social media, only those who knew how to use technology and had access to data could respond. This explains why younger women and university graduates were over-represented among our respondents. Further, we used PLAFAM's social media pages to promote the survey, and PLAFAM is the only entity in Venezuela that has been able to provide reliable and affordable access to contraceptive methods in recent years. Hence our high prevalence of contraceptive use is likely a biased view that is only representative of PLAFAM's clientele and not of the Venezuelan population as a whole. That said, most of our respondents experienced fuel shortages, lack of access to the Internet, clean water, medicines, and transportation, among other challenges – thus demonstrating that they were not insulated by privilege. Social desirability bias is another risk inherent to any survey that covers sensitive topics related to sexual and reproductive health – it is possible that respondents under-reported abortions, for example (16). However, the anonymity of our survey may have reduced this bias.

Our study also has several strengths. First, our survey was designed and pilot-tested with input from multiple stakeholders in Venezuela and around the world. Second, it utilizes a novel strategy, harnessing the power of social media to reach a wide range of women who would not otherwise be engaged in research. It is important to note that the study was conducted in July of 2020, at the height of the COVID-19 pandemic. Social media allowed us to reach a relatively large number of respondents remotely without putting their health at risk. Finally, this is the only study to our knowledge that quantitatively describes women's access to abortion and contraception in the country's humanitarian context, providing a snapshot of the two-year period from July 2018 to July 2020. Overall, our findings represent a new contribution to the scientific literature. Future research should continue to investigate the impact on women's reproductive and sexual health of the evolving Venezuelan political and economic context.

## Data Availability

The raw data supporting the conclusions of this article will be made available by the authors, without undue reservation.
